# Predictors of Hospital Mortality and the Related Burden of Disease in Severe Traumatic Brain Injury: A Prospective Multicentric Study in Brazil

**DOI:** 10.3389/fneur.2019.00432

**Published:** 2019-04-25

**Authors:** Fernando Zanela Areas, Marcelo Liborio Schwarzbold, Alexandre Paim Diaz, Igor Kunze Rodrigues, Daniel Santos Sousa, Camila Leite Ferreira, João Quevedo, Katia Lin, Emil Kupek, Cristiane Ritter, Felipe Dal Pizzol, Roger Walz

**Affiliations:** ^1^Centro de Neurociências Aplicadas, Universidade Federal de Santa Catarina, Hospital Universitário, Florianópolis, Brazil; ^2^Programa de Pós-Graduação em Neurociências, UFSC, Florianópolis, Brazil; ^3^Programa de Pós-Graduação em Ciências Médicas, UFSC, Florianópolis, Brazil; ^4^Serviço de Psiquiatria, Departamento de Clínica Médica, HU, UFSC, Florianópolis, Brazil; ^5^Serviço de Neurocirurgia, HU, UFSC, Florianópolis, Brazil; ^6^Serviço de Neurocirurgia, Hospital Regional de São José Homero de Miranda Gomes, São José, Brazil; ^7^Serviço de Neurocirurgia, Hospital Governado Celso Ramos, Florianópolis, Brazil; ^8^Laboratório de Neurociências, Programa de Pós-Graduação em Ciências da Saúde, Universidade do Extremo Sul Catarinense, Criciúma, Brazil; ^9^Department of Psychiatry and Behavioral Sciences McGovern Medical School, The University of Texas Health Science Center at Houston, Houston, TX, United States; ^10^Serviço de Neurologia, Departamento de Clínica Médica, HU, UFSC, Florianópolis, Brazil; ^11^Departmento de Saúde Pública, UFSC, Florianópolis, Brazil; ^12^Hospital São José, Criciúma, Brazil; ^13^Laboratório de Fisiopatologia Experimental, Programa de Pós-Graduação em Ciências da Saúde, UNESC, Criciúma, Brazil

**Keywords:** traumatic brain injury, mortality, prognosis, burden of disease, Brazil

## Abstract

Traumatic brain injury (TBI) is a worldwide social, economic, and health problem related to premature death and long-term disabilities. There were no prospective and multicentric studies analyzing the predictors of TBI related mortality and estimating the burden of TBI in Brazil. To address this gap, we investigated prospectively: (1) the hospital mortality and its determinants in patients admitted with severe TBI we analyzed in three reference centers; (2) the burden of TBI estimated by the years of life lost (YLLs) due to premature death based on the hospital mortality considering the hospital mortality. Between April 2014 and January 2016 (22 months), all the 266 patients admitted with Glasgow coma scale (GCS), ≤ 8 admitted in three TBI reference centers were included in the study. These centers cover a population of 1,527,378 population of the Santa Catarina state, Southern Brazil. Most patients were male (*n* = 230, 86.5%), with a mean (SD) age of 38 (17) years. Hospital mortality was 31.1% (*n* = 83) and independently associated with older age, worse cranial CT injury by the Marshall classification, the presence of subarachnoid hemorrhage in the CT, lower GCS scores and abnormal pupils at admission. The final multiple logistic regression model including these variables showed an overall accuracy for hospital mortality of 77.9% (specificity 88.6%, sensitivity 53.8%, PPV 67.7%, and NPV 81.1%). The estimated annual incidence of hospitalizations and mortality due to severe TBI were 9.5 cases and 5.43 per 100,000 inhabitants, respectively. The estimated YLLs in 22 months, in the 2 metropolitan areas were 2,841, corresponding to 1,550 YLLs per year and 101.5 YLLs per 100,000 people every year. The hospital mortality did not change significantly since the end of the 1990s and was similar to other centers in Brazil and Latin America. Significant predictors of hospital mortality were the same as those of studies worldwide, but their strength of association seemed to differ according to countries income. Present study results question the extrapolation of TBI hospital mortality models for high income to lower- and middle-income countries and therefore have implications for TBI multicentric trials including countries with different income levels.

## Introduction

Traumatic brain injury (TBI) is a worldwide social, economic, and health problem related to premature death and long-term disabilities (1–5). Depending on the TBI severity and its complications, even in the absence of motor impairment, the TBI may result in cognitive deficits (6, 7), endocrine (8) and psychiatric disorders (9–11) affecting work capacity (12), and impairing the quality of life (9).

Based on their Gross National Income per capita value, the World Bank classified the countries as having lower, middle, or high income. It is believed the incidence and mortality of TBI are higher in lower- and middle-income countries (LMIC) than in high-income countries (HIC) (5, 13, 14). Differences in the rescue time and the medical care may result in a higher proportion of patients surviving with a disability in HIC in comparison to LMIC (13). Interestingly, the proportion of survivors patients remaining with disability following a severe TBI seems to be similar between LMIC and HIC, and patients LMIC shows better recovery after a mild or moderate TBI. The reduced levels of disability in LMIC may be partly explained by excess mortality following severe TBI, as well by the differences in the definition of disability between settings, socio-cultural and environmental particularities which affect how society interprets and reacts to disability (13).

In Brazil, like in several other LMIC, the TBI related mortality and morbidity are not systematically recorded and only two studies evaluated prospectively the hospital mortality of patients with TBI and determined its independent predictors (15, 16) In our previous single center, 748 patients from Florianópolis, the Santa Catarina State capital (Southern Brazil), admitted with coma Glasgow scale (GCS) ≤ 8 were included between January 1994 and December 2003 (16). Another Brazilian study included patients admitted at the emergency unit of the University of São Paulo Hospital with an admission GCS score who had an abnormal brain computerized tomography (CT) scan (*n* = 1,275) between September 2003 and December 2009. The older age, lower GCS, worse cranial Computed Tomography (CT) lesions by Marshall CT classification and the pupillary abnormality remained as independent predictors of hospital mortality in both studies. These predictors are associated with mortality and in TBI patients worldwide, although the strength of the association may differ according to the country's income level (3, 13). In both studies, the binary regression models showed a global accuracy for hospital mortality of 77%.

Diagnostic and therapeutic decisions are based on the patient's prognosis. Prognostic models are statistical models that combine two or more variables of patient's data to predict clinical outcome. There were no prospective and multicentric studies analyzing the predictors of TBI related mortality and estimating the burden of TBI in Brazil. To address this gap, we investigated prospectively: (1) the hospital mortality and its determinants in patients admitted with severe TBI we analyzed in three reference centers; (2) the burden of TBI estimated by the years of life lost (YLLs) due to premature death based on the hospital mortality considering the hospital mortality, the life expectancy, as well as the population covered by the TBI reference centers during the study period.

## Methods

### Patients

All patients with severe TBI admitted to the intensive care unities of three reference hospitals for brain trauma in the public health system of in Santa Catarina state, southern Brazil, between April 2014 and January 2016 (22 months) were included in this study. The Hospital Governador Celso Ramos (HGCR) in the city of Florianópolis and the Hospital Homero de Miranda Gomes (HHMG) in the city of São José cover the Greater Florianópolis metropolitan area with the population of 940,935 inhabitants. The Hospital São José (HSJ) in the city Criciúma, which covers another metropolitan area with 586,443 inhabitants also participate in this study. The population was determined by the official Brazilian Census Survey of 2014. Both metropolitan areas are crossed by the busiest national Brazilian highway (called BR-101), with a mean daily local traffic of 175,000 vehicles.

Inclusion criteria were GCS score 8 or lower after acute neurosurgical resuscitation, without sedation, or deterioration to that level within 48 h of the hospital admission. The same research protocol design was used in previous studies (6, 16–19).

### Hospital Mortality, Predictive Variables, and Burden of TBI

The primary endpoint was in-hospital mortality. The demographic, clinical, neuroradiological, and neurosurgical analyzed variables were: gender, age, cause of TBI, Marshall classification of cranial CT, intracranial pressure monitoring (ICP), decompressive craniectomy, presence of subarachnoid hemorrhage (SAH) as evidenced by CT, associated trauma, admission GCS, admission pupils' response, reference trauma center, injury severity score (ISS), systolic and diastolic blood pressure at ICU admission, glucose, sodium, hemoglobin, and leucocyte at the ICU admission. These variables were included in the research protocol based on previous studies of other authors and our experience in severe TBI research (3, 4, 6–12, 16). All the CT scans were evaluated by the neurosurgeon and the ICU specialist. A decompressive craniectomy was done only in 13 (5%) patients, according to the personal decision of the neurosurgeon and other particularities including the age, hemodynamic state, the presence of multiple traumas. They had hemispheric edema and midline shift > 5 mm, diffuse brain edema with a refractory elevation of intracranial pressure, or when the neurosurgeon has the impression that the brain remains tense even after the mass lesions were evacuated.

To estimate the burden related to severe TBI we did the sum of the years of life lost (YLLs) due to premature mortality (20, 21) among patients who died during the hospitalization. We also estimated the cumulative YLLs per year. Because in Brazil virtually all cases of TBI are treated by the public health system at the reference centers for trauma, it was possible to estimate the YLLs per year per 100,000 inhabitants. The life expectancy reported by the Brazilian Institute of Geography and Statistics (IBGE) in the state of Santa Catarina in 2017 was 72.5 year for men and 79.6 years for women. The burden of severe TBI was determined according to the formulas below:

YLLs per patient = Life expectancy – age of death due to severe TBI

Cumulative YLLs in 22 months = ∑ (YLLs patient 1 + …. + YLLs patient 83

YLLs per year = (Cumulative YLLs in 22 months / 22) x 12

YLLs per year per 100,000 inhabitants = [YLLs per year / (Population)] x 100,000.

### Statistical Analysis

After a descriptive analysis of patients' characteristics, we did a univariate binary logistic regression to identify the variables associated with hospital mortality with a type I error significance level ≤ 0.20 (see Table 1). These variables were included in the multiple binary regression model using a forward conditional method to determine the independent predictors of hospital mortality. Because the univariate analysis showed that Marshall III and Evacuated mass showed the same prognosis, they were categorized together in the multiple regression analysis. The ISS score was arbitrarily categorized using the mean ISS score of patients as the cut-off. The glucose levels were arbitrarily divided into 3 categories. Leukocytosis was defined as more than 11.000 leucocytes/mm^3^, and there were no patients with leukopenia in our sample. The magnitude of the association between the outcome and the independent variables was measured by the odds ratio (OR) and respective 95% confidence interval (CI). A type I error ≤ 0.05 was considered statistically significant. No adjustment for multiple comparisons was made because the independent variables included had a clinical relevance. The model discrimination was assessed by the area under the ROC curve generated using the predicted probabilities from the model. Statistical analysis was performed using the SPSS program 20.0 (Chicago, IL).

**Table 1:** Distribution of clinical, demographic, radiological, and neurosurgical variables of patients admitted with severe traumatic brain injury according to the hospital mortality.

**Variables**	**All patients *n* = 266 (%)**	**Outcome**		**Crude OR (CI 95%)**	**p value**
		**Survivors *n* = 183 (68.5%)**	**Death *n* = 83 (31.1%)**	**for hospital mortality**	
**Gender**					
Male	230 (86.5)	162 (70.4)	68 (29.6)	1.0	
Female	36 (13.5)	21 (58.3)	15 (41.7)	1.70 (0.83–3.50)	0.15
**Age (years)^a^**					
Mean (± SD)	38 (17)	37.8 (16.8)	39.7 (17.4)	N.A.	0.39
18 to 34 years	138 (51.9)	98 (71.0)	40 (29.0)	1.0	
35 to 65 years	105 (39.5)	73 (69.5)	32 (30.5)	1.07 (0.62–1.87)	0.80
Older than 65 years	23 (8.6)	12 (52.2)	11 (47.8)	2.25 (0.91–5.51)	0.08
**Cause of TBI^b^**					
Physical aggression	17 (6.4)	13 (76.5)	04 (23.5)	1.0	
Car accident	37 (13.9)	23 (62.2)	14 (37.8)	1.98 (0.54–7.28)	0.30
Pedestrian trampling	33 (12.4)	23 (69.7)	10 (30.3)	1.41 (0.37–5.42)	0.61
Bicycle accident	04 (1.5)	02 (50.0)	02 (50.0)	3.25 (0.34–31.07)	0.31
Falls	57 (21.4)	40 (70.2)	17 (29.8)	1.38 (0.39–4.85)	0.61
Gunshot	12 (4.5)	07 (58.3)	05 (41.7)	2.32 (0.47–11.54)	0.30
Motorcycle accident	94 (35.2)	67 (71.3)	27 (28.7)	1.31 (0.39–4.38)	0.66
Unknown^c^	12 (4.5)	08 (66.7)	04 (33.3)	1.62 (0.31–8.39)	0.56
**Marshall CT classification^d^**					
Type I injury	18 (6.8)	17 (94.4)	01 (5.6)	1.0	
Type II injury	51 (19.2)	45 (88.2)	06 (11.8)	2.27 (0.25–20.24)	0.46
Type III injury	96 (36.1)	63 (65.6)	33 (34.4)	8.90 (1.13–69.89)	0.04
Type IV injury	47 (17.7)	18 (38.3)	29 (61.7)	27.40 (3.35–223.84)	0.002
Evacuated mass lesion	48 (18.0)	35 (72.9)	13 (27.1)	6.31 (0.76–52.33)	0.09
**ICP Monitoring^e^**					
No	201 (76.7)	141 (70.1)	60 (29.9)	1.0	
Yes	61 (23.3)	39 (63.9)	22 (36.1)	1.32 (0.72–2.42)	0.36
**Decompressive craniectomy^e^**					
No	249 (95.0)	170 (68.3)	79 (31.7)	1.0	
Yes	13 (5.0)	10 (76.9)	03 (23.1)	0.65 (0.17–2.41)	0.52
**SAH on CT Scan^f^**					
No	135 (50.8)	106 (78.5)	29 (21.5)	1.0	
Yes	123 (46.2)	73 (59.3)	50 (40.7)	2.50 (1.45–4.32)	0.001
**Associated trauma**					
No	109 (41.0)	70 (64.2)	39 (35.8)	1.0	
Yes	157 (59.0)	113 (72.0)	44 (28.0)	0.70 (0.41–1.18)	0.18
**Admission glasgow coma scale**					
7 or 8	104 (39.1)	90 (86.5)	14 (13.5)	1.0	
5 or 6	31 (11.7)	21 (67.7)	10 (32.3)	3.06 (1.19–7.83)	0.02
3 or 4	131 (49.2)	72 (55.0)	59 (45.0)	5.27 (2.72–10.19)	<0.0001
**Admission pupillary response^g^**					
Isocoric	192 (72.2)	145 (75.5)	47 (24.5)	1.0	
Anisocoric	52 (19.5)	31 (59.6)	21 (40.4)	2.09 (1.10–3.98)	0.02
Bilateral mydriasis	18 (98.5)	05 (27.8)	13 (72.2)	8.02 (2.72–23.68)	<0.0001
**Reference trauma center^h^**					
HGCR	83 (31.3)	56 (68.3)	26 (31.7)	1.0	
HHMG	123 (46.1)	88 (71.5)	35 (28.5)	0.86 (0.47–1.57)	0.62
HSJ	61 (22.8)	39 (63.9)	22 (36.1)	1.21 (0.60–2.45)	0.58
**Injury severity score, mean (SD)^i^**	21.0 (9.9)	19.5 (9.5)	24.7 (10.0)	N.A.	0.001
<21	109 (55.6)	32 (29.4)	77 (70.6)	1.0	
≥22	87 (44.4)	46 (52.9)	41 (47.1)	2.56 (1.34–4.87)	0.004
**Systolic BP, ICU admission, mmHg^j^**	128.4 (23.6)	129.2 (23.0)	126.6 (25.0)	N.A.	0.41
**Diastolic BP, ICU admission, mmHg^k^**	73.1 (16.3)	73.1 (15.7)	73.0 (17.9)	N.A.	0.93
**Glucose, ICU Admission, mg/dL^l^**					
Mean (±SD)	142.8 (42.5)				0.01
≤ 110	37 (13.9)	33 (89.2)	04 (10.8)	1.0	
111 to 160	139 (52.3)	91 (65.5)	48 (34.5)	4.35 (1.46–13.00)	0.008
>160	14.0 (7.4)	07 (50.0)	50.0 (7.7)	8.25 (1.89–36.04)	0.005
**Sodium, ICU admission, mEq/L^m^**	139.4 (11.3)	140.5 (9.7)	140.8 (9.4)	N.A.	0.89
**HB, ICU admission, gm/dL^n^**	11.3 (4.7)	11.0 (1.9)	11.2 (2.9)	N.A.	0.66
**Leucocytes/mm^3^, ICU admission^o^**	13969 (5216)	135589 (4969)	14880 (5553)	NA	0.07
≤ 11.000	72 (27.1)	26 (36.1)	31 (43.1)	1.0	
>11.000	167 (62.8)	45 (26.9)	76 (45.5)	1.86 (0.98–3.53)	0.06

a *Age was not confirmed in 06 cases (2.2%)*.

b *The cause of trauma was unknown 3 cases (1.1%)*.

c *Unknown, patients found with TBI but without a clear history of the cause of TBI*.

d *Tomographic Marshall scale was not evaluated in 6 patients (2.2%)*.

e *Information about intra-cranial pressure (ICP) monitoring or decompressive craniectomy procedure was not available in four patients*.

f *SAH = Sub-arachnoids hemorrhage not evaluated in 8 cases (3.0%)*.

g *Four patients (1.5%) had ocular trauma and pupils were not adequately evaluated*.

h *Trauma Centers: HGCR, Hospital Governador Celso Ramos; HHMG, Hospital Homero de Miranda Gomes; HSJ, Hospital São José*.

i *ISS was not determined in 70 patients (26.3%). The 21 ISS score the upper band of the 95% CI for the mean ISS of the survivors and the lower band of the 95% CI for the mean ISS of non-survivors*.

j *Systolic BP, Systolic blood pressure at the intensive care unit (ICU) admission expressed in mmHg*.

k *Diastolic BP, Diastolic blood pressure at ICU admission expressed in mmHg*.

l *Glucose levels at the ICU admission were not determined in 76 patients (28.6%) expressed in mg/dL*.

m *Sodium levels at the ICU admission were not determined at ICU admission in 106 patients (39.8%) expressed in mEq/dL*.

n *Hemoglobin levels at the ICU admission were not determined in 15 patients (5.6%) expressed in gm/dL*.

o *Leucocytes levels per mm^3^ at the ICU admission was not determined in 27 patients (10.1%)*.

The probability of death for an individual patient can be calculated based on the formula including the respective B coefficient for each category of the variables showed in the final regression model (see Table 2) using the formula below:

**Table 2:** The clinical, demographic, radiological, and neurosurgical variables independently associated with a hospital mortality of patients admitted with severe traumatic brain injury.

**Predictive variables**	**B coefficient**	**Adjusted OR (CI 95%) for hospital mortality**	***“p” value***
**Constant**	−3.91		<0.0001
**Age^a^**			
Younger than 65 years old		1.0	
65 years or older	1.74	5.48 (1.87–16.10)	0.002
**Marshall CT classification^b^**			
Type I		1.0	
Type II	0.10	1.11 (0.11–11.03)	0.93
Type III or V	0.96	2.60 (0.30–22.36)	0.38
Type IV	2.20	9.04 (1.00–81.41)	0.05
**SAH on the CT Scan**			
No		1.0	
Yes	1.06	2.87 (1.47–5.63)	0.001
**GCS**			
7 or 8		1.0	
5 or 6	1.50	4.45 (1.47–14.50)	0.008
3 or 4	1.73	5.60 (2.43–12.89)	<0.0001
**Pupillary**			
Isocoric		1.0	
Anisocoric	0.21	1.24 (0.58–2.65)	0.58
Bilateral mydriasis	1.25	3.48 (1.03–11.67)	0.04
**Model accuracy**			
*The area under de ROC*			0.82
*Overall model accuracy*			77.9%
*Specificity*			88.6%
*Sensitivity*			53.8%
*Positive predictive value*			67.7%
*Negative predictive value*			81.1%

*The initial multiple binary regression model included only the patients (n = 253) in which all predictive variables were available at the ICU admission. Variables included in the initial model were: gender, age, Marshall CT classification, SAH on CT scan, associated trauma, Glasgow coma scale (GCS), pupil's examination. There were 175 survivors (69.2%) and 78 non-survivors (n = 30.8%)*.

a *Patients were divided into 2 categories according to their ages*.

b *Marshall Type III injury and evacuated mass lesion were combined in one category*.

Probability of death = 1 / (1 + e^−z^)

Where:

e = Euler's number = 2.7182818284590452 (and more…)

z = B of the constant + Age category * (B for age) + Marshall CT category * (B for Marshall) + GCS category * (B for GCS category) + Pupillary response category * (B for pupillary category).

Probability of death = 1 / (1 + e^−z^)

Where:

e = Euler's number = 2.7182818284590452 (and more…)

z = B of the constant + variable *A* category ^*^ (B for variable *A* category) + variable B category ^*^ (B for variable *B* category) + variable *C* category ^*^ (B for variable *C* category) + variable *D* category ^*^ (B for variable *D* category)

B = Coefficients of the binary regression, for the constant and for each category of the variables included in the final binary regression model.

## Results

There were 266 hospitalizations due to severe TBI resulting in 83 (31.1%) deaths during the study period of 22 months. Considering the population of 1,527,378 inhabitants covered by the participating centers, the hospitalization rate was 9.5 cases per 100,000 habitants per year and the mortality was 5.43 per 100,000 habitants per year. During the study period, there were 2,841 YLLs, corresponding to 1,550 YLLs per year and 101.5 YLLs per 100,000 people per year in the metropolitan areas analyzed.

Table 1 shows the distribution of demographic, clinical, radiological and neurosurgical characteristics of the patients admitted with severe TBI according to their hospital mortality. Hospital mortality was not associated with the TBI etiology, ICP monitoring, reference trauma center, and systemic blood pressure, sodium, hemoglobin levels at the ICU admission (*p* > 0.20). There was a trend for lower hospital mortality among patients with associated trauma other than TBI (*p* = 0.18), as well for increased hospital mortality among females (*p* = 0.15), older ages (*p* = 0.08), and leukocytosis at ICU admission (*p* = 0.06). The mortality was significantly higher among patients with worse injury on the CT scan according to Marshall CT classification (*p* < 0.05), presence of SAH on CT scan (*p* < 0.001), lower GCS score at ICU admission (*p* < 0.0001), higher ISS on ICU admission (*p* = 0.004), and higher glucose levels at ICU admission (*p* < 0.01).

Table 2 shows the clinical, demographic, radiological, and neurosurgical variables independently associated with hospital mortality, as determined by the binary multiple regression analysis. Older age, worse lesion on Marshall CT classification, the presence of SAH, lower GCS scores and abnormal pupils' examination at ICU admission remained independently associated with the hospital mortality. The final model showed an overall accuracy of 77%, with a specificity of 88.5%, and a sensitivity of 53%. Because the gunshot wounds constitute a separate population from blunt injury, we did a multiple binary regression using the variables shown in Table 2 and including the cause of TBI, attributing 1 to the OR for death by physical aggression. The accuracy of the model remains the same. There was a non-significant trend for higher mortality in the severe TBI caused by gunshot (Adjusted OR 4.3, CI 95% 0.6 to 33.3, *p* = 0.15) than physical aggression (data not shown).

We also ran a multiple binary logistic regression model including the variables gender, age, Marshall CT classification, SAH on CT scan, pupils' examination, injury severity score (ISS), GCS, glucose levels, and leukocytes levels at ICU admission. For this analysis, 113 patients had all these predictive variables available at the ICU admission. The age, SAH on CT scan, pupil's examination and ISS score (instead of GCS) remained in the model which showed an overall accuracy of 77%, with a specificity of 90.2%, and a sensitivity of 41.9% (details not shown).

## Discussion

This is the first prospective, a multicentric study assessing hospital mortality due to severe TBI in Brazil. The results were similar to our (16) and others (15, 22) previous single-center studies in Brazil and multicentric studies in Latin America (14, 23). The hospital mortality in the present study was similar in the three centers analyzed (range between 28.5 to 36.1%) and did not change significantly in comparison to the mortality registered in the city of Florianópolis, at the HGCR, between 1994 and 2003 (16). A large Chinese retrospective TBI study (*n* = 7,145) from 47 hospitals recruited between 1 December 2008 and 20 August 2009 showed a 21.8% hospital mortality among the 1626 (22.8%) patients admitted with GCS ≤ 8, which was closer to the observed in HIC and lower than in Brazil and other LMIC (24). Although the differences in the medical care between HIC and LMIC countries may exist, we believe the delay between the accident and the effective rescue and hospitalization is an important factor that contributes to the higher mortality of our patients with severe TBI in our center in comparison to HIC. Unfortunately, during the course of this study, we did not obtain reliable information to investigate this hypothesis.

We show that older age, Marshall CT classification, presence SAH on the CT scan, lover GCS scores, and pupillary abnormalities were independent predictors of hospital mortality. Our results are in agreement with the International Mission on Prognosis and Analysis of Clinical Trials in TBI study (IMPACT) (25) The regression model including these variables showed a similar accuracy (77.9%) to predict the hospital mortality as two previous prospective studies conducted at the HGCR in the city of Florianópolis between 1994 and 2003 (16) and at the São Paulo university hospital between 2003 and 2009 (15). The lower mortality observed in São Paulo compared to the present study may be due to the inclusion of some patients with higher GCS scores (range between 3 and 15) as opposed to the patients with severe TBI (GCS ≤ 8) in the present study. In accordance to the São Paulo study (15), we did not confirm our previous study evidencing the association between the concomitant thoracic trauma and lower hospital mortality (16), and we have none plausible explanation for this conflicting results. Interestingly, although hormonal (18), immunologic (19), or biochemical markers (17) in the blood collected during the acute phase of severe TBI has been associated to hospital mortality and sometimes even improved the prognostic models, the age, pupillary examination and GCS score at admission always remained independently associated with hospital mortality in our previous studies (17–19). This indicated the consistency of these variables to predict hospital mortality in our population.

Based on the regression model described by Junior et al. in the aforementioned São Paulo study (15), the probability of death during hospitalization in a Brazilian patient with 65-year-old age, with a GCS of 7, a Marshall CT classification of 4, and isochoric pupils is estimated to be around 47.7%. Based on the present study, the estimated mean probability for hospital death for the patient described above was about 50.7%. For this analysis, subarachnoid hemorrhage in the CT was considered absent because this variable was not analyzed in the São Paulo study. However, if the same patient had a CT with subarachnoid hemorrhage the probability of death would rise to 75% in our model. For comparison, we also applied the well-known CRASH model to the same hypothetical Brazilian patient above. Because in our protocol we did not evaluate the presence of petechial hemorrhages and no obliteration of the third ventricle or basal cisterns, we run the CRASH model in the absence of both variables, which results in 46%. Including petechial hemorrhages, the risk increases to 52.5% and if the patients also had obliteration of the third ventricle or basal cisterns the risk became 68.7%. Because the CRASH model is applicable to patients up to 8 h after the accident, the reader should be aware of the lack of confirmation of the time course between the accident and hospitalization in our sample.

In the present study, the burden of severe TBI was estimated by the YLLs metrics based on hospital mortality. This approach did not take into account patients who died after the hospitalization, resulting in an underestimation of the YLLs in our sample. Based on our clinical observation, we believe the mortality in the first year after the hospitalization can be up to 15%, but this remains to be confirmed by further empirical studies. One randomized, prospective, multicentric trial on intracranial-pressure monitoring (23) in Bolivia and Ecuador (*n* = 324) shows a 14-day mortality of 25.5% which increased to 40% in the 6 months after hospitalization.

The reader should be aware of some the limitations of our study: (i) the sample was comprised by patients with severe TBI and admitted to a reference trauma facility, which may limit generalizability to non-reference center; (ii) the lower number of cases and variability in decision criteria for decompressive craniectomy does not allow to establish acceptable conclusions about the impact of this therapeutic modality on hospital mortality of our patients; (iii) the outcome was restricted to hospital mortality; (iv) the burden of TBI was estimated based on the hospital mortality and not the disability-adjusted life years (DALYs); (v) because 2 of the 3 hospitals involved in this study are references for trauma occurring at BR-101, the busiest Brazilian highway that runs through diverse metropolitan areas, some of the included patients maybe were not living in the cities of the metropolitan areas used to estimate the population in risk for severe TBI. Nevertheless, the strengths of our study are: (i) the prospective and multicentric study design; (ii) acute patients with severe TBI in the geographic areas of the study are almost exclusively assisted by the three participating reference centers, implying the inclusion of virtually all severe TBI patients in these regions during the period of study; (iii) this is the first prospective study estimating the disease burden of TBI in Brazilian patients.

## Conclusions

This is the first multicentric and prospective study on hospital mortality and the burden of TBI based on YLLs in Brazil. The burden of TBI determined by disability-adjusted life years (DALYs) remains to be investigated in Brazil. Older age, Marshall CT classification, presence SAH on the CT scan, lower GCS scores, and pupillary abnormalities are independent predictors of hospital mortality. The hospital mortality due to severe TBI was similar to those from other trauma centers in Brazil and Latin America and did not change significantly since the end of the 1990s. Present study findings are in agreement with the view that extrapolation from predictive TBI models in HIC to LMIC may lead to inaccurate estimation of TBI-related hospital mortality. These results have implications for further TBI multicentric trials including countries with different income levels.

## Ethics Statement

This study was carried out in accordance with the recommendations of Comitê de Ética em Pesquisa em Seres Humanos da Universidade (Plataforma Brazil Registration 02832612.6.1001.0121). Informed consent to participate was signed by each patient's family.

## Author Contributions

AD, JQ, KL, EK, FD, and RW: study design, literature revision, manuscript revision. RW and FD: statistical analysis and manuscript writing. FA, IR, MS, DS, CF, CR, and FD: data collection, and manuscript revision.

### Conflict of Interest Statement

The authors declare that the research was conducted in the absence of any commercial or financial relationships that could be construed as a potential conflict of interest.
